# Iterative Screening Density Peak Clustering

**DOI:** 10.3390/e28101155

**Published:** 2026-10-20

**Authors:** Zilong Deng, Mustafa Muwafak Alobaedy, Shahid Kamal, Fang Cheng

**Affiliations:** 1 Centre for Image and Vision Computing, CoE for Artificial Intelligence, Faculty of Computing and Informatics, Multimedia University, Cyberjaya 63100, Selangor, Malaysia; dzlong@aqvtc.edu.cn; 2 College of Information Technology, Anqing Vocational and Technical College, Anqing 246003, China; 3 Center for Advanced Analytics, CoE for Artificial Intelligence, Faculty of Computing & Informatics, Multimedia University, Cyberjaya 63100, Selangor, Malaysia; shahid.kamal@mmu.edu.my

**Keywords:** density peak, screening, image clustering

## Abstract

Density peak clustering (DPC) is popular in unsupervised clustering due to its intuitive theory and the absence of pre-defined cluster numbers. However, traditional DPC relies on a static single computation strategy. It uses all raw samples to calculate global density and relative distance without iterative correction. Low-density samples in real data distort the density distribution, weaken the peak characteristics of real clustering centers, lead to incorrect center selection and disordered point allocation, and damage the stability and accuracy of clustering. To address this limitation, we propose an Iterative Screening Density Peak Clustering (IS-DPC). A new local density based on binary selection is designed to suppress interference from low-density samples at the computational source. In addition, the iterative mechanism transforms static single-round calculations into dynamic progressive updates. Experiments on real datasets have shown that IS-DPC outperforms several advanced DPC variants in most cases.

## 1. Introduction

Rodriguez and Laio proposed density peak clustering (DPC), which has been widely used in data mining [1]. However, traditional DPC follows a static one-time computation framework [2]. It uses all original samples to simultaneously calculate global density, relative distance, and select cluster centers, without correction in the subsequent calculation process. The actual data collected from real-world scenarios usually contains low-density samples [3], which weaken the peak features of natural clustering centers, leading to incorrect estimation of the number of clusters and chaotic allocation of data points [4]. The inherent errors caused by static computational structures cannot be automatically repaired, which greatly limits the clustering stability and accuracy of DPC on real datasets.

In order to address the core drawback of easily obtaining inappropriate clustering centers through static DPC, this paper proposes an Iterative Screening Density Peak Clustering (IS-DPC) algorithm. We introduce a new local density based on binary selection to shield low-density samples and isolate noise interference during density calculation. By combining a multi-round iterative strategy with gradually increasing density thresholds, the original one-time static calculation is reconstructed into a dynamic progressive optimization process. In each iteration, the density distribution and scoring metrics of the cluster centers are updated based on the purified sample set obtained from previous screening. The numerical difference between the real clustering centers and the normal samples continues to widen to avoid the generation of false centers, and optimized clustering results are derived after iterative convergence.

The main contributions of this paper are summarized into two aspects:

1. Constructing a binary selection for improving local density. The 0-1 mask matrix eliminates the interference of low-density samples from the density summation. The suppression of density distribution distortion caused by abnormal data at the source of numerical calculation has solved the problem of low clustering center resolution caused by mixed global data calculation in the original DPC.
2. Establish an iterative progressive optimization mechanism. Transform the traditional static one-time clustering solution framework into a multi-round iterative update architecture. Purify the sample space layer by layer through a slowly increasing density threshold, iteratively correcting the evaluation coefficients of density, relative distance, and cluster centers. It can accurately identify the real cluster centers, significantly reducing the probability of selecting false centers incorrectly.

## 2. Density Peak Clustering (DPC)

DPC was proposed by Rodriguez and Laio in 2014 [5], and it is based on two basic assumptions: the cluster center itself has a high local density. The distance between the centers of different clusters is relatively far. DPC calculates two feature quantities: the local density ρ (the density of surrounding data) and relative distance δ (the distance between data points that are closest to the point and have a higher density than it) for each data point [6].

DPC draws a decision graph with δ as the vertical axis and ρ as the horizontal axis, the points with high ρ and high δ are the cluster centers of each cluster, and users need to manually select cluster centers from the decision graph.

According to the order of density from high to low, non-center points are directly assigned to the clusters of the closest and densest classified samples, and all sample classifications are completed at once [7].

There is no iterative operation in DPC, and clustering partitioning is completed solely based on the relationship between distance and density.

## 3. Review on DPC

Scholars have proposed numerous improved DPC variants, and the improved methods can be divided into the following branches.

### 3.1. Improve the Density Calculation of DPC

This category reconstructs the local density calculation formula and reduces the distortion of density distribution caused by isolated noises and abnormal boundary points.

Wan et al. combined natural neighbor with a multi-cluster merging strategy to suppress the interference of outliers in the density calculation process [8]. Xie et al. improved the recognition accuracy of real density peaks under a noisy environment [9]. Xie et al. further designed WANN-DPC with a weighted adaptive nearest neighbor to realize differentiated density solutions for dense and sparse areas of the data distribution [10]. Ding et al. defined weighted KNN local density to replace the original Gaussian kernel density [11]. Waqas et al. introduced a Gaussian fuzzy neighborhood with a noise control parameter to enhance the anti-interference ability of density evaluation [12]. Chen et al. utilized mutual nearest neighbor distance to revise density measurement [13]. In addition, three-way DPC realizes noise marking and density correction for incomplete datasets [14].

### 3.2. Automatic DPC

DPC has parameters and needs human to select cluster centers on decision graph, which is not convenient for users.

Guan et al. proposed a peak-padding mechanism with minimum cost to calibrate deviated density peaks and realize automatic peak screening without manual intervention [15]. Zang et al. constructed DPC-MFP relying on multiple feature points to adaptively match reasonable cluster centers for complex data [16]. Zhang et al. adopted neural membrane migration rules to build a self-organized density peak searching model [17]. Zhang et al. excavated stable cluster centers via support point and nearest neighbor topological relation [18].

### 3.3. Improve the Sample Assignment Strategy

Some research optimizes label allocation rules or carries out redundant sub-cluster merging after clustering to cut off error transmission paths.

Li et al. proposed DPC-DNG, which replaced the original simple assignment rule with a graph topology constraint [19]. Xie et al. built a cost-aware clustering framework to optimize the allocation of samples [20]. Gao et al. designed a cross-view confidence constraint to optimize sample division [21]. Granular ball-based DPC optimizes boundary sample assignment [22,23].

### 3.4. Reduce Time Complexity

One branch focuses on reducing the quadratic time complexity of original DPC. Ma et al. proposed UP-DPC ultra-scalable parallel DPC [24]; Zheng et al. accelerated neighbor searching and density computation using a grid-block partitioning mechanism [25]; and Guo et al. constructed a hybrid clustering framework based on improved DPC to balance clustering accuracy and calculation overhead [26]. Sieranoja et al. used a k-nearest-neighbor graph to estimate local density and relative distance [27].

### 3.5. Variable Cut-Off Threshold

The original DPC employs a global fixed cutoff distance $d_{c}$ determined by a fixed percentage of pairwise distances, which may yield unsatisfactory performance for datasets with highly heterogeneous density distribution. To address this drawback, several studies focus on developing variable cut-off threshold schemes for DPC [28]. Instead of adopting a uniform global distance percentage, the K-nearest-neighbor-based DPC with aggregating strategy calculates local density using locally varied distance scales across different data regions. The Delaunay-graph-based DPC further introduces region-dependent cut-off thresholds according to topological properties of data distribution [29]. Meta-heuristic algorithms are also utilized to search for proper threshold parameters. For example, the improved black-widow optimization is adopted to tune threshold-related hyperparameters [30].

### 3.6. Outlier Removal

Outlier and noise suppression is a critical topic for clustering. Some improved DPC algorithms perform outlier filtering as a preprocessing stage before clustering. For instance, weighted mutual *K*-nearest-neighbor-based DPC filters potential outliers relying on neighbor-weighted statistics to reduce noise interferenc [31].

### 3.7. Research Gap

Unlike existing static weighted density or one-time noise filtering methods, this paper introduces a 0-1 binary selection to participate in density accumulation calculation. Low-density samples are masked as zero weight and excluded from the density solving process, avoiding the density distortion problem caused by the global mixing calculation of clean and abnormal samples in traditional improved DPC.

In addition, compared with outlier filtering, as in [32], we acknowledge that both follow the core principle of iterative filtering.

Nevertheless, the two methods differ in the stage at which noise is handled. The research objective of [32] is to remove noise (or its effect in clustering); the chosen method applies mean-shift iteratively, and it uses KNN to estimate density. The authors of [32] execute independent outlier filtering as a standalone pre-step ahead of the real clustering algorithm. Noise samples are detected and filtered before density calculation and cluster-center searching. In contrast, our IS-DPC embeds noise suppression inside the iterative clustering loop.

## 4. Algorithm Procedures of IS-DPC

To provide a clearer explanation of our algorithm, we will first provide symbol definitions (see Table 1).

### 4.1. Data Normalization

For the original data matrix X, we use Z-score normalization:

(1) $${\tilde{x}}_{ij}=\frac{x_{ij}-\mu_{j}}{\sigma_{j}},$$

where $\mu_{j}$ and $\sigma_{j}$ represent the mean value and standard deviation of the *j*-th feature column respectively.

The range of different feature values in the original data varies greatly, and after normalization, the contribution weights of each feature to the distance are equal. The calculated local density of the samples can truly reflect the spatial clustering distribution of the data and will not be disturbed by the numerical range of the original data.

Then, we calculate the global cutoff distance $d_{c}$, compute pairwise Euclidean distances $∥{\tilde{x}}_{i}-{\tilde{x}}_{j}{∥}_{2}\;\left(i<j\right)$ among all samples, sort all distances in ascending order, and compute the truncation position: $pos=\left\lfloor \frac{n(n-1)}{2}\cdot dc_{\mathrm{ratio}}\right\rfloor$ ($dc_{\mathrm{ratio}}$ is a user-defined parameter), assigning $d_{c}$ to the value located at position pos in the sorted distance sequence.

### 4.2. Iterative Screening

The original DPC only calculates the Gaussian density once using all the original data. Discrete low-density and peripheral outlier samples can distort the true spatial clustering distribution: the peak density at the center of the cluster is smoothed out, and the density at the boundary points is abnormally raised, making it easy to misidentify low-density as the cluster center or miss the true cluster center. The high-density core points, edge transition points, and external low-density points within the cluster are mixed together to participate in the operation, which makes the clustering division highly prone to misclassification.

Therefore, we propose an iterative screening mechanism: progressive data cleaning, refining the sample set from coarse to fine, and ultimately retaining the core sample subset with a high clustering degree within each cluster. The iterative process will continuously strengthen and widen the density peak at the center of the cluster compared to ordinary samples, making the density distribution more in line with the true cluster structure of the data.

For the iteration *t*, we calculate the weighted local density $\rho_{i}^{\left(t\right)}$:

(2) $$\rho_{i}^{\left(t\right)}=\sum_{j=1}^{n}v_{j}^{\left(t-1\right)}\exp\left(-\frac{∥{\tilde{x}}_{i}-{\tilde{x}}_{j}{∥}_{2}^{2}}{d_{c}^{2}}\right).$$

When calculating the original global density $\rho_{i}$, low-density points are scattered around the periphery of the cluster, which can increase the density of normal samples in the periphery and decrease the density advantage of the cluster center, resulting in a distorted density distribution. Thus, we propose the addition of binary selection $v_{j}^{\left(t-1\right)}$, low-quality samples are directly removed from the summation term, and the density is only calculated from clean cluster ontology samples. The density value can truly reflect the degree of data aggregation.

The binary selection $v_{j}^{\left(t\right)}$ is defined as

(3) $$v_{i}^{\left(t\right)}=\left\{\begin{array}{cc} 1, & \rho_{i}^{\left(t\right)}>\lambda^{\left(t-1\right)}\\ 0, & \rho_{i}^{\left(t\right)}\leq \lambda^{\left(t-1\right)} \end{array}\right.,\;\lambda^{\left(t\right)}=\lambda^{\left(t-1\right)}+\Delta \lambda .$$

Obviously, $v_{j}\in \left\{0,1\right\}$ in our design; if $v_{j}=1$, the sample is a valid high-quality sample retained by the screening process in the previous iteration and participates in density calculation, whereas if $v_{j}=0$, low-density invalid samples are masked and excluded from density accumulation.

In the original global density calculation, scattered low-density points are distributed around clusters. Such low densities will raise the density values of surrounding normal samples and weaken the density predominance of cluster centers, resulting in a distorted density distribution. After introducing binary selection, low-quality samples are directly removed from the summation terms. The density is calculated only using pure samples inside clusters, such that the solved density values can faithfully reflect the actual aggregation level of data.

The algorithm gradually increases the density threshold λ to eliminate low-quality samples step by step. The density is recalculated in each iteration using the sample subset screened in the last iteration. In the first iteration, calculate density with all samples to roughly filter out obvious abnormal points. In the subsequent iterations, recalculate density based on the purified subset, so the density distribution fits the real cluster structure better and better.

This is a coarse-to-fine progressive optimization strategy, which cannot be realized via one-time standalone calculation.

We calculate relative distance $\delta_{i}^{\left(t\right)}$ in the iteration *t*; if $S_{i}\neq \emptyset$ ($S_{i}$ denotes a set of samples satisfying $\rho_{j}^{\left(t\right)}>\rho_{i}^{\left(t\right)}$),

(4) $$\delta_{i}^{\left(t\right)}=\min_{j\in S_{i}}{∥{\tilde{x}}_{i}-{\tilde{x}}_{j}∥}_{2},$$

Otherwise, $S_{i}=\emptyset$, whose $\delta_{i}^{\left(t\right)}$ is assigned the maximum value among all finite δ values.

At last, we can obtain the cluster center score γ:

(5) $$\gamma_{i}^{\left(t\right)}=\rho_{i}^{\left(t\right)}\cdot \delta_{i}^{\left(t\right)}.$$

We sort γ values in descending order, and select the indices of the top-*k* values to form the cluster center set *C*, initialize the label vector $Y_{\mathrm{pred}}^{\left(t\right)}=-1\cdot 1_{n}$, and  assign exclusive labels to each cluster center, traversing all unlabeled samples in descending order of $\rho_{i}^{\left(t\right)}$. For sample *i*, inherit the label from the nearest sample that is already labeled and has higher density, and map the original messy center indices to consecutive natural numbers to standardize label encoding.

After finishing all *T* iterations, take $Y_{\mathrm{pred}}^{\left(T\right)}$ as the final clustering labels.

In order to better understand our proposed algorithm, we have provided pseudocode (see Algorithm 1).

IS-DPC has three parameters: $dc_{\mathrm{ratio}}$, $\lambda_{0}$ and *k*.

### 4.3. Time Complexity Analysis

The time complexity of vanilla DPC is $O(n^{2})$, dominated by pairwise distance calculation. For the proposed IS-DPC, local density and relative distance are recomputed over retained core samples in each iteration. Given the maximum iteration number *T*, the worst-case asymptotic complexity yields: $O(Tn^{2})$. This corresponds to the scenario that few samples are filtered during iterations. Although the asymptotic order remains quadratic, the iterative mechanism brings considerably larger constant factors and higher practical runtime compared with standard DPC.
**Algorithm 1** Iterative Screening Density Peak Clustering (IS-DPC)**Require:** Original dataset matrix $X\in R^{n\times d}$, maximum iteration number *T*, initial density       threshold $\lambda_{0}$, threshold increment Δλ, proportional factor $dc_{\mathrm{ratio}}$, ground truth labels       $Y_{\mathrm{true}}$
**Ensure:** Final clustering label vector $Y_{\mathrm{pred}}^{\left(T\right)}$, clustering evaluation metrics ARI and NMI
  1:Normalize original data via Z-score standardization: $\tilde{X}\leftarrow Z\text{-}\mathrm{score}\left(X\right)$  2:Calculate all pairwise Euclidean distances between samples  3:Compute the position index: $pos=\left\lfloor \frac{n(n-1)}{2}\cdot dc_{\mathrm{ratio}}\right\rfloor$  4:Sort the distance sequence in ascending order, assign $d_{c}=\mathrm{sortedDist}\left[pos\right]$ as the Gaussian kernel cutoff distance  5:Initialize sample mask vector: $v^{\left(0\right)}=1_{n}$  6:Initialize original density threshold: $\lambda^{\left(0\right)}=\lambda_{0}$  7:**for** t=1 **to** *T* **do**  8:    Compute new local density based on binary selection for each sample:  9:    **for** i=1 **to** *n* **do**$$\rho_{i}^{\left(t\right)}=\sum_{j=1}^{n}v_{j}^{\left(t-1\right)}\exp\left(-\frac{{∥{\tilde{x}}_{i}-{\tilde{x}}_{j}∥}_{2}^{2}}{d_{c}^{2}}\right)$$10:    **end for**11:    Update density threshold: $\lambda^{\left(t\right)}=\lambda^{\left(t-1\right)}+\Delta \lambda$12:    Update screening mask to eliminate noise and outliers:13:    **for** i=1 **to** *n* **do**$$v_{i}^{\left(t\right)}=\left\{\begin{array}{cc} 1, & \rho_{i}^{\left(t\right)}>\lambda^{\left(t-1\right)}\\ 0, & \rho_{i}^{\left(t\right)}\leq \lambda^{\left(t-1\right)} \end{array}\right.$$14:    **end for**15:    Calculate the relative distance $\delta_{i}^{\left(t\right)}$ utilizing screened valid samples16:    Compute comprehensive score for cluster center selection: $\gamma_{i}^{\left(t\right)}=\rho_{i}^{\left(t\right)}\cdot \delta_{i}^{\left(t\right)},\forall i\in \left[1,n\right]$17:    Select *k* cluster centers according to the descending order of $\gamma_{i}^{\left(t\right)}$18:    Assign cluster labels to obtain temporary clustering result $Y_{\mathrm{pred}}^{\left(t\right)}$19:**end for**20:Calculate ARI, NMI, and ACC with $Y_{\mathrm{pred}}^{\left(T\right)}$ and ground truth $Y_{\mathrm{true}}$21:**return** $Y_{\mathrm{pred}}^{\left(T\right)}$, ARI, NMI, and ACC

## 5. Experiments and Discussions

The experimental hardware is a laptop equipped with an Intel i7-1750H CPU and 16 GB RAM.

Two mainstream clustering evaluation metrics are used to quantitatively measure clustering performance, namely the Adjusted Rand Index (ARI), Normalized Mutual Information (NMI), and ACC. The range of these two indicators is between 0 and 1. A larger value indicates higher consistency between the predicted partition and the ground truth labels, which means better clustering quality. To show the reality of our results, we upload the ARI, NMI, and ACC codes to our GitHub (https://github.com/DENGZILONG0712/IHS-DPC/tree/main, accessed on 28 September 2026).

### 5.1. Datasets Description

In this paper, nine widely used clustering benchmark datasets covering small low-dimensional tabular data, medium feature datasets and high-dimensional image data are adopted to evaluate the performance of all algorithms. The datasets include Seeds, Wine, Heart, Banknote, Landsat, MSRA25, Palmdata, USPS and PenDigits. Among them, Seeds, Wine, Heart and Banknote are classic low-dimensional UCI datasets with overlapping cluster boundaries, which are suitable for verifying the ability of density clustering to distinguish overlapping samples. Landsat belongs to medium-dimensional remote sensing feature data with complex sample distribution. MSRA25, Palmdata, USPS and PenDigits are high-dimensional handwritten or face image datasets, which are used to test the scalability and feature capture capability of each clustering method. Data and codes are available at https://github.com/DENGZILONG0712/IHS-DPC, accessed on 19 August 2026. The detailed statistical information of all datasets is summarized in Table 2.

### 5.2. Baselines

We compare the proposed IS-DPC with five state-of-the-art density peak clustering baselines, namely FDPAN [33], RMDPC [34], DBSCAN-DPC [35], DPCSA [36] and SMMP [37]. The core ideas and hyperparameter configurations of each competitor are elaborated as follows:

1. **FDPAN** [33]: Fast density peak adaptive neighbor clustering. Its adjustable hyperparameters are denoted as (numAnchor/k). numAnchor stands for the number of anchor points sampled to reduce computational cost, and *k* represents the maximum number of adaptive neighbors for each sample. We perform grid search over the parameter space and record the optimal metrics for each dataset.
2. **RMDPC**: Robust main density peak clustering. It eliminates fake cluster peaks caused by outliers via local region constraints. No manual hyperparameters are required, marked as “/” in the table.
3. **DBSCAN-DPC** [35]: A hybrid density clustering algorithm combining DBSCAN connectivity and density peak screening. Its hyperparameter group is written as (nk/nsmaple/minpts). nk is the number of k-nearest neighbors for distance statistics; nsmaple denotes the scaling coefficient of the neighborhood distance threshold; minpts is the minimum number of adjacent samples to judge core points in DBSCAN.
4. **DPCSA** [36]: Density peak clustering based on weighted local density sequence. It corrects density bias from a single cutoff distance and selects cluster centers automatically without adjustable hyperparameters, denoted as “/”.
5. **SMMP** [37]: Stable-membership multi-peak clustering, which filters redundant density peaks through multi-layer membership matrices. All thresholds are self-adaptive with no tunable parameters.

For all comparative algorithms, we conduct a full grid search on their hyperparameter spaces and record the ARI and NMI values with optimal parameter settings in Table 3, Table 4 and Table 5.

The proposed IS-DPC contains three tunable hyperparameters, namely $dc_{\mathrm{ratio}}$, $\lambda_{0}$ and *k*, whose specific physical meanings and functions are explained as follows:

1. ${\mathrm{dc}}_{\mathrm{ratio}}$: Cutoff distance ratio. It is the proportional coefficient used to calculate the local density cutoff distance. The actual cutoff distance $d_{c}$ is determined by sorting all pairwise sample distances and taking the quantile corresponding to $dc_{\mathrm{ratio}}$.
2. $\lambda_{0}$: Initial density threshold.
3. k: Number of final clusters. This hyperparameter specifies the preset total number of clustering categories for the dataset. After screening density peaks via adaptive density estimation, the algorithm selects the top *k* samples with comprehensive high density and large separation distance as global cluster centers to complete the final sample assignment.

In our experiments, we traverse a unified grid search range for the three hyperparameters and select the group with the highest ARI and NMI values as the optimal configuration reported in Table 3, Table 4 and Table 5.

### 5.3. Comparison of Real-World Datasets

We compare the proposed IS-DPC with five state-of-the-art density peak clustering methods including FDPAN [33], RMDPC [34], DBSCAN-DPC [35], DPCSA [36] and SMMP [37]. Nine benchmarks covering low-dimensional data and high-dimensional image data are utilized to comprehensively verify the clustering accuracy of all methods based on the ARI and NMI values shown in Table 3, Table 4 and Table 5.

For the Heart dataset, IS-DPC achieves the best ACC of 0.80, while its ARI is only 0.35 and NMI is 0.28. The ARI values of other competing algorithms are generally lower than 0.16. For MSRA25, IS-DPC does not achieve optimal results across all metrics: SMMP yields considerably better ARI (0.64) and NMI (0.87) than IS-DPC (ARI = 0.38, NMI = 0.69); IS-DPC obtains an ACC of 0.57, which is only tied with RMDPC. For the remaining datasets, IS-DPC outperforms most competing algorithms.

For some results, ari and acc demonstrate big differences from nmi, but we do not know the reason. We offer the predicted label vectors and upload them to GitHub, where everyone can use them to calculate the ari, nmi, and acc.

According to previous research results [38], classification-oriented datasets should generally be used with caution when directly evaluating clustering algorithms [39]. Therefore, we further adopt synthetic datasets to verify the performance of the proposed algorithm.

### 5.4. Comparison of Synthetic Datasets

We use seven controlled synthetic clustering datasets including Aggregation, R15, D31, S1, S4, A3, and Unbalance [40], as described in [38], to evaluate the correctness of sample assignment, the ACC metric is adopted. Thus, in this paper, we use three metrics including ARI, NMI, and ACC for a more comprehensive evaluation of algorithm performance [39].

See the clustering results in Table 6; the performance of the proposed algorithm remains competitive. Our method has some advantages: it performs better on datasets R15, D31, S1, S4, and A3, but it fails on Aggregation and Unbalance.

**Figure 1:**
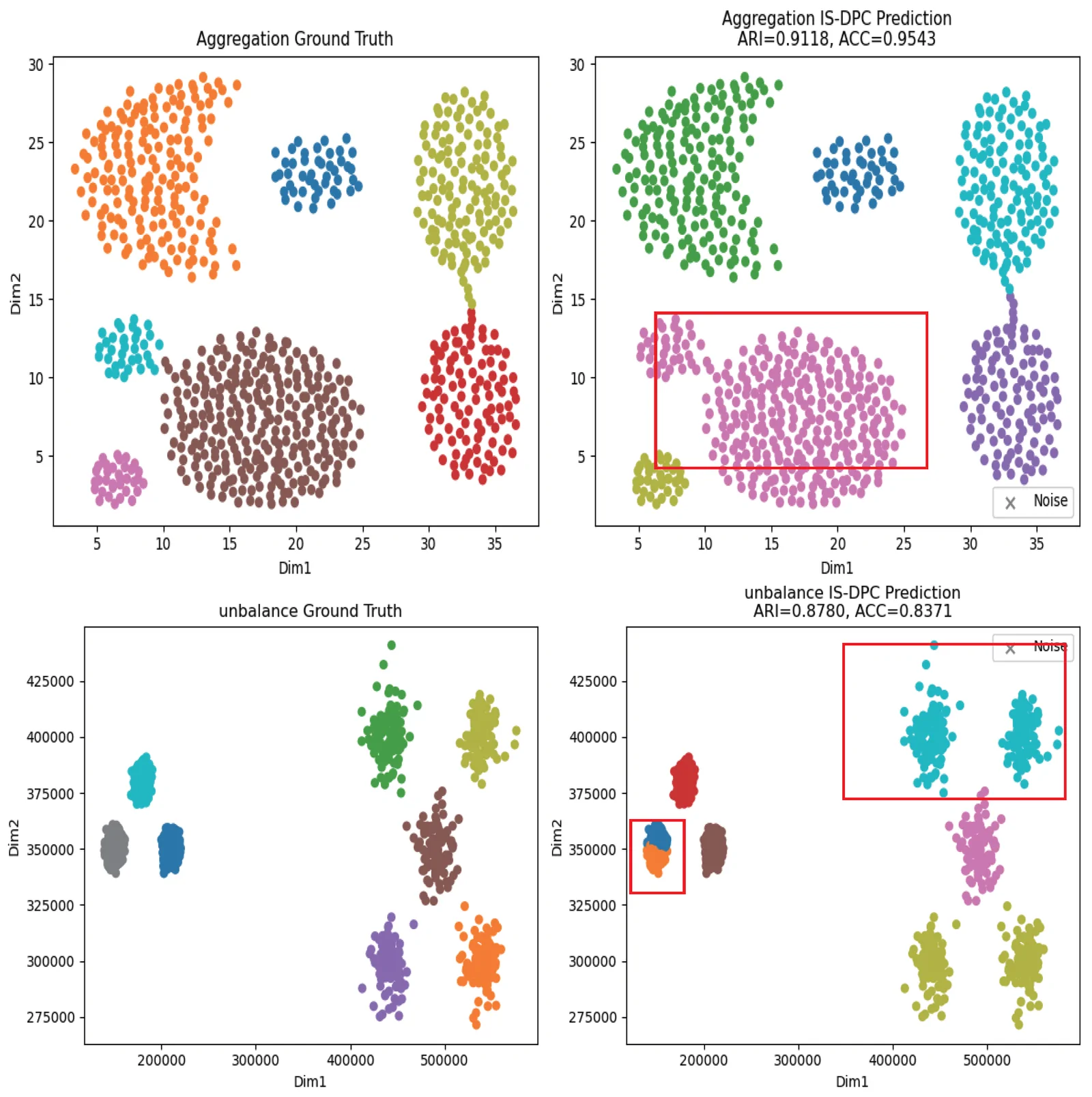
Clustering visualization results of synthetic datasets, different colors represent different clusters.

See the results of Aggregation in Figure 1; the true classes in the rectangle have only one density peak with higher local density, which is selected as one center. This reason leads to low accuracy. The basic reason is that Aggregation is an unbalanced dataset; the basic algorithm in our method is density peak clustering (the core part uses DPC for clustering), which cannot deal with this situation. Additionally, if one class has one density peak with high local density, density peak-based clustering algorithms favor this situation.

For the dataset Unbalance, the cluster within the left rectangle has high local density; two clusters exist in one true class. See the right rectangle, where two classes with lower local density have one density peak, and they are merged into one cluster.

In addition, the dataset Birch has 100,000 points, our method has an out-of-memory error.

### 5.5. Ablation Study

**Figure 2:**
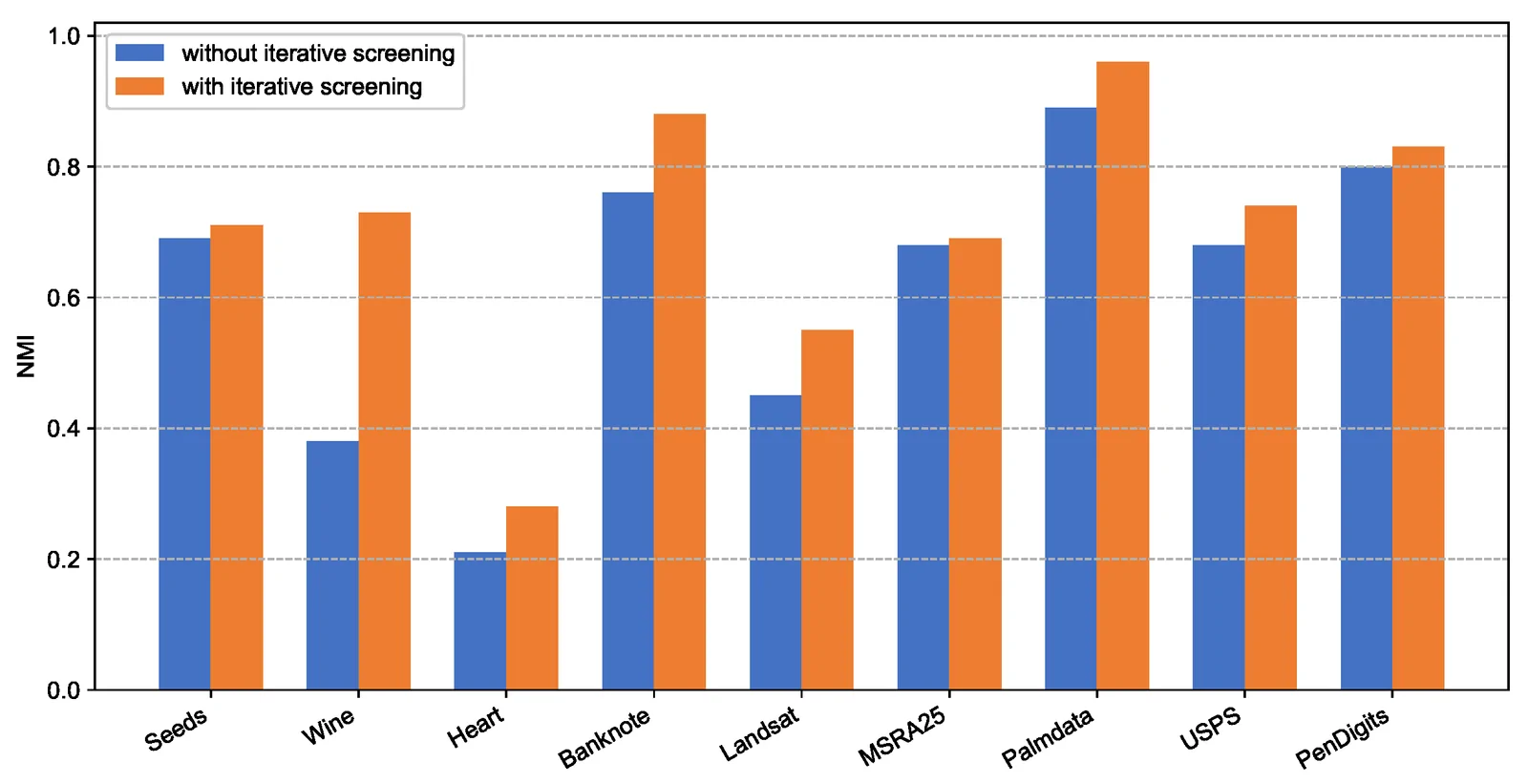
Ablation study of IS-DPC.

The NMI results of the ablation study are illustrated in Figure 2. We delete the iterative screening and compare it with full IS-DPC; after removing the iterative screening mechanism, the clustering accuracy decreases on all datasets.

This observation can be explained by our mechanism: the binary mask gradually masks out low-density unreliable samples during each iteration. Only high-confidence core samples are used to recalculate local density, relative distance, and cluster center score. This progressively amplifies the density gap between real cluster centers and ordinary points, suppressing false peaks.

We use [32] to remove noise, then use IS-DPC or the original DPC to group the remaining data and assign noise to the nearest clusters. We call them MOS-ISDPC or MOS-DPC.

See Table 7, except for the Unbalance dataset; the clustering performance degrades on all the datasets after introducing the noise elimination module [32]. MOS is valid for such unbalanced datasets. The performance of MOS-ISDPC is close to that of MOS-DPC. This is because MOS has pre-filtered most outliers and noisy samples, so the iterative hard-screening module of IS-DPC loses its room to work.

### 5.6. Application of Face Clustering

**Figure 3:**
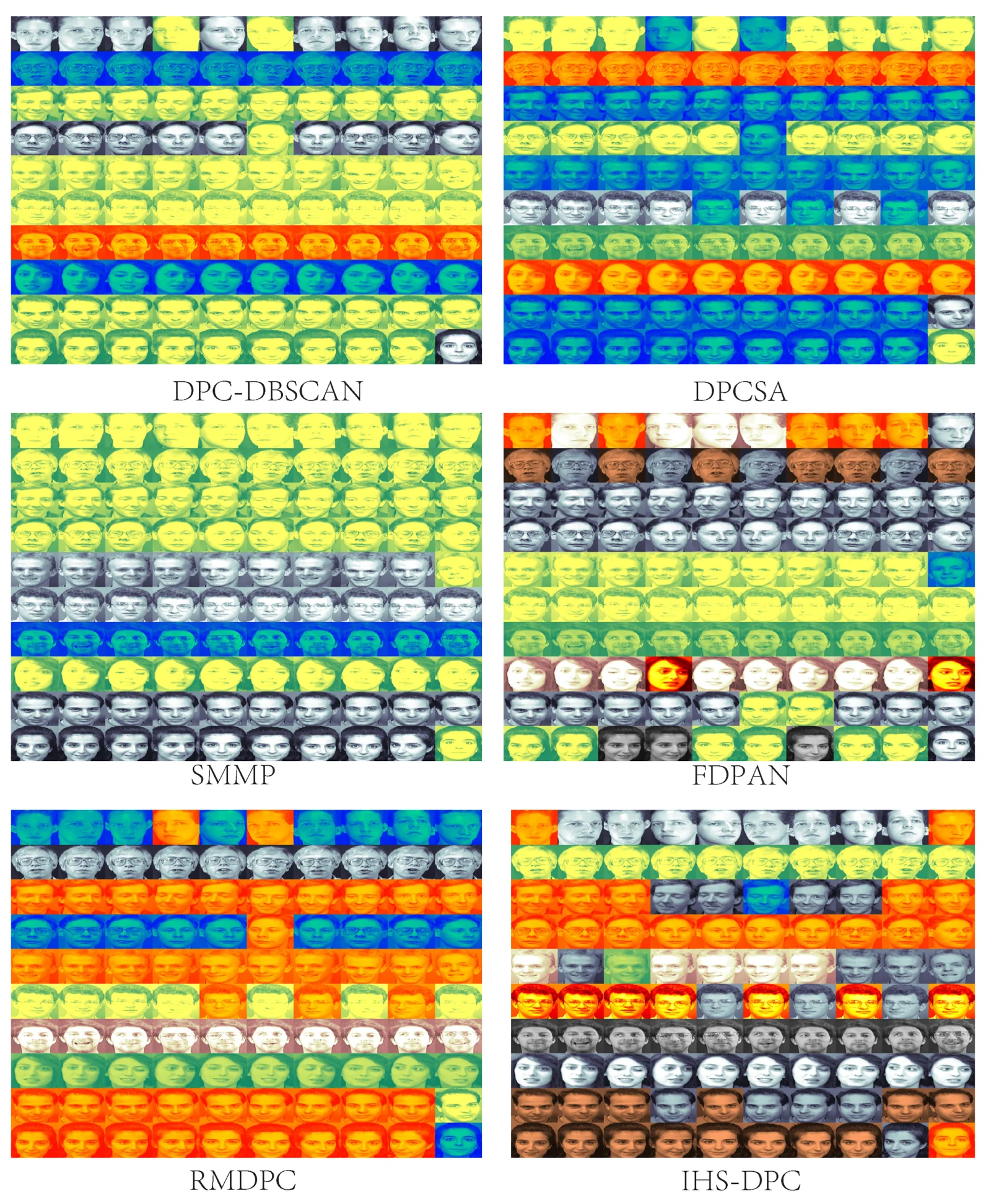
Clustering visualization results of different algorithms on the Olivetti Face dataset.

Experiments were conducted on the Olivetti Face dataset using Adjusted Rand Index (ARI) and Normalized Mutual Information (NMI) as evaluation metrics (see Table 8). It can be observed that the proposed IS-DPC achieves optimal performance at ARI = 0.5267 and NMI = 0.7509. Compared with several improved clustering methods based on density peaks such as FDPAN, RMDPC, DPCSA, DPC-DBSCAN, and SMMP, IS-DPC achieved better results for both indicators, which verifies its stronger clustering ability for face datasets. In addition, we also provided visualized clustering results (see Figure 3).

In IS-DPC, the binary selection gradually masks out low-density unreliable samples during each iteration. Only high-confidence core samples are used to recalculate local density, relative distance and cluster center score. This progressively amplifies the density gap between real cluster centers and ordinary points, suppressing false peaks. The performance gain is data-dependent: prominent improvements are observed for datasets contaminated with abundant low-density samples the such as Olivetti face dataset, while marginal gains are obtained for compact, well-separated datasets.

### 5.7. Parameter Tuning

In unlabeled real-world scenarios where ground-truth labels are unavailable, hyperparameters cannot be optimized via clustering metrics, such as the ARI or NMI. For $dc_{ratio}$, we tune this parameter by observing the decision graph of γ values. A proper $dc_{ratio}$ should yield a decision graph with several obviously outstanding high-γ points, which correspond to potential cluster centers. If γ values are too crowded without prominent peaks, we increase $dc_{ratio}$; if almost all points show negligible γ, we decrease $dc_{ratio}$.

For the initial screening threshold $\lambda_{0}$, we refer to the quantile statistics of the initial local density distribution. We monitor the number of valid retained samples in each iteration. If valid samples drop sharply in early iterations, we reduce $\lambda_{0}$ to relax screening. If few samples are filtered out throughout iterations, we raise $\lambda_{0}$ for stronger noise suppression.

For cluster number *k*, after iterative convergence, we plot the decision graph based on final-iteration γ scores. Similar to vanilla DPC, we select prominent high-γ points as cluster centers to determine *k* without ground-truth labels.

Moreover, we clarify the termination principle of our algorithm: instead of adaptive convergence judgment, our algorithm adopts a fixed maximum iteration number *T* as the stopping criterion in this work.

The hyperparameter search ranges for grid search are as follows: $dc_{ratio}\in \left[0.01,0.05\right]$. Once set, it does not change. The initial value $\lambda_{0}\in \left[0.01,0.22\right]$, $\lambda^{\left(t\right)}=\lambda^{\left(t-1\right)}+\Delta \lambda$, Δλ=0.1. In addition, we often set the *k* to the true number of clusters.

## 6. Conclusions

In response to the shortcomings of traditional DPC, including static single computation, density distortion caused by low-density samples, and frequent erroneous cluster center selection, this work has developed two key improvements: a new local density calculation and an iterative mechanism. Unlike existing DPC variants that rely on static weighting, one-time noise filtering, or post-processing correction, IS-DPC dynamically purifies samples throughout the clustering pipeline. The introduced 0-1 mask matrix eliminates anomalous samples in the density summation process.

The experiments on nine benchmarks ranging from low-dimensional table data to high-dimensional images have demonstrated that IS-DPC performs better than five advanced DPCs in most cases. Further validation of the Olivetti Face dataset indicates that IS-DPC performs the best. The visualization results confirm its ability to distinguish subtle facial features. In summary, IS-DPC overcomes the limitations of static computation in traditional DPC, enhances robustness to outliers, and provides an effective unsupervised clustering solution for general data mining and image analysis tasks.

Future research can be conducted considering four aspects. Firstly, the automatic estimation of the number of clusters is integrated to eliminate dependence on the preset parameter *k*. Secondly, an adaptive update strategy for the threshold increment Δλ is designed to replace the fixed step size. Thirdly, approximate neighborhood search or sampling methods are adopted to reduce the computational overhead caused by repeated density calculations, thus enabling the processing of large-scale datasets.

## Figures and Tables

**Table 1:** Symbol Definitions

Mathematical Symbol	Definition
$X=\{x_{ij}\}$	Original feature matrix
*n*	Total number of samples
*d*	Feature dimension per sample
$\tilde{X}=\left\{{\tilde{x}}_{ij}\right\}$	Z-score-normalized feature matrix for distance and density calculation
${\tilde{x}}_{i}$	Normalized feature vector of the *i*-th sample
$Y_{\mathrm{true}}$	Ground-truth labels for clustering evaluation only
$Y_{\mathrm{pred}}^{\left(t\right)}$	Predicted clustering labels at iteration *t*; $Y_{\mathrm{pred}}^{\left(T\right)}$ denotes the final output labels
*k*	Real number of clustering categories
${∥\cdot ∥}_{2}$	L2 norm for computing Euclidean distance between samples
$dc_{\mathrm{ratio}}$	Scale coefficient for truncating sorted distance sequence (hyperparameter)
$d_{c}$	Global fixed cutoff distance of Gaussian kernel
*T*	Total iteration count of the algorithm, constant T=20
*t*	Current iteration number, t=1,2,…,T
Δλ	Increment step of screening threshold per iteration, constant Δλ=0.1
$\lambda_{0}$	Initial value of density screening threshold (hyperparameter)
$v^{\left(t\right)}$	Sample screening mask at iteration *t*; $v_{i}^{\left(t\right)}=1$ means sample is retained, $v_{i}^{\left(t\right)}=0$ means sample is discarded
$\lambda^{\left(t\right)}$	Density screening threshold used in iteration *t*, with initial condition $\lambda^{\left(0\right)}=\lambda_{0}$
$\rho_{i}^{\left(t\right)}$	Local density of the *i*-th sample at iteration *t*
$\delta_{i}^{\left(t\right)}$	Relative distance of the *i*-th sample at iteration *t*
$S_{i}$	Index set of samples satisfying $\rho_{j}^{\left(t\right)}>\rho_{i}^{\left(t\right)}$
$\gamma_{i}^{\left(t\right)}$	Comprehensive score for cluster center evaluation, calculated as $\gamma_{i}^{\left(t\right)}=\rho_{i}^{\left(t\right)}\cdot \delta_{i}^{\left(t\right)}$
*C*	Index set of selected cluster centers
$N_{\mathrm{valid}}^{\left(t\right)}$	Quantity of valid samples preserved by the mask at iteration *t*
$K_{\mathrm{cur}}^{\left(t\right)}$	Actual effective cluster quantity obtained at iteration *t*

**Table 2:** Statistical information of nine benchmark datasets.

Dataset	Number of Samples	Feature Dimension	Number of Categories
Seeds	210	7	3
Wine	178	13	3
Heart	303	13	2
Banknote	1372	4	2
Landsat	6435	36	6
MSRA25	2000	128	25
Palmdata	2000	128	10
USPS	11,000	256	10
PenDigits	10,992	16	10

**Table 3:** ARI comparison of six methods. Values in parentheses after metrics denote parameters.

Algorithm	Seeds	Wine	Heart	Banknote
FDPAN	0.34(5/4)	0.42(5/5)	0.16(5/5)	0.88(5/5)
RMDPC	0.62(/)	0.50(/)	0.07(/)	0.41(/)
DBSCAN-DPC	0.44(11/0.028/4)	0.28(11/0.028/4)	0.11(11/0.028/4)	0.11(11/0.028/4)
DPCSA	0.62(/)	0.39(/)	0.03(/)	0.52(/)
SMMP	0.53(/)	0.45(/)	0.00(/)	0.63(/)
IS-DPC	0.77(0.032/0.14/3)	0.70(0.026/0.19/3)	0.35(0.025/0.18/2)	0.92(0.025/0.18/2)
**Algorithm**	**Landsat**	**MSRA25**	**Palmdata**	**USPS**
FDPAN	0.29(4/5)	0.37(5/5)	0.62(9/5)	0.43(5/10)
RMDPC	0.38(/)	0.37(/)	0.01(/)	0.07(/)
DBSCAN-DPC	0.35(11/0.028/4)	0.31(11/0.028/4)	0.70(11/0.028/4)	0.40(11/0.028/4)
DPCSA	0.27(/)	0.32(/)	0.32(/)	0.12(/)
SMMP	0.07(/)	0.64(/)	0.00(/)	0.27(/)
IS-DPC	0.44(0.025/0.18/6)	0.38(0.021/0.21/25)	0.82(0.024/0.20/10)	0.64(0.025/0.18/10)
**Algorithm**	**Data_PenDigits**			
FDPAN	0.74(5/4)			
RMDPC	0.65(/)			
DBSCAN-DPC	0.68(11/0.028/4)			
DPCSA	0.72(/)			
SMMP	0.70(/)			
IS-DPC	0.75(0.023/0.19/10)			

**Table 4:** NMI comparison of six methods. Values in parentheses after metrics denote parameters.

Algorithm	Seeds	Wine	Heart	Banknote
FDPAN	0.44(5/4)	0.54(5/5)	0.12(5/5)	0.83(5/5)
RMDPC	0.61(/)	0.56(/)	0.10(/)	0.57(/)
DBSCAN-DPC	0.55(11/0.028/4)	0.38(11/0.028/4)	0.11(11/0.028/4)	0.46(11/0.028/4)
DPCSA	0.64(/)	0.56(/)	0.18(/)	0.61(/)
SMMP	0.57(/)	0.58(/)	0.10(/)	0.67(/)
IS-DPC	0.71(0.032/0.14/3)	0.73(0.026/0.19/3)	0.28(0.025/0.18/2)	0.88(0.025/0.18/2)
**Algorithm**	**Landsat**	**MSRA25**	**Palmdata**	**USPS**
FDPAN	0.39(4/5)	0.68(5/5)	0.90(9/5)	0.29(5/10)
RMDPC	0.46(/)	0.68(/)	0.27(/)	0.31(/)
DBSCAN-DPC	0.40(11/0.028/4)	0.60(11/0.028/4)	0.83(11/0.028/4)	0.58(11/0.028/4)
DPCSA	0.38(/)	0.65(/)	0.42(/)	0.32(/)
SMMP	0.24(/)	0.87(/)	0.14(/)	0.56(/)
IS-DPC	0.55(0.025/0.18/6)	0.69(0.021/0.21/25)	0.96(0.024/0.20/10)	0.74(0.025/0.18/10)
FDPAN	0.82(5/4)			
RMDPC	0.77(/)			
DBSCAN-DPC	0.78(11/0.028/4)			
DPCSA	0.69(/)			
SMMP	0.80(/)			
IS-DPC	0.83(0.023/0.19/10)			

**Table 5:** ACC comparison of six methods. Values in parentheses after metrics denote parameters.

Algorithm	Seeds	Wine	Heart	Banknote
FDPAN	0.56(5/4)	0.66(5/5)	0.70(5/5)	0.96(5/5)
RMDPC	0.75(/)	0.39(/)	0.29(/)	0.48(/)
DBSCAN-DPC	0.61(11/0.028/4)	0.57(11/0.028/4)	0.57(11/0.028/4)	0.18(11/0.028/4)
DPCSA	0.73(/)	0.54(/)	0.51(/)	0.67(/)
SMMP	0.67(/)	0.63(/)	0.14(/)	0.79(/)
IS-DPC	0.92(0.032/0.14/3)	0.89(0.026/0.19/3)	0.80(0.025/0.18/2)	0.98(0.025/0.18/2)
**Algorithm**	**Landsat**	**MSRA25**	**Palmdata**	**USPS**
FDPAN	0.46(4/5)	0.47(5/5)	0.72(9/5)	0.64(5/10)
RMDPC	0.73(/)	0.57(/)	0.03(/)	0.12(/)
DBSCAN-DPC	0.66(11/0.028/4)	0.51(11/0.028/4)	0.40(11/0.028/4)	0.37(11/0.028/4)
DPCSA	0.46(/)	0.41(/)	0.51(/)	0.41(/)
SMMP	0.32(/)	0.58(/)	0.04(/)	0.53(/)
IS-DPC	0.67(0.025/0.18/6)	0.57(0.021/0.21/25)	0.84(0.024/0.20/10)	0.75(0.025/0.18/10)
**Algorithm**	**Data_PenDigits**			
FDPAN	0.92(5/4)			
RMDPC	0.93(/)			
DBSCAN-DPC	0.79(11/0.028/4)			
DPCSA	0.76(/)			
SMMP	0.84(/)			
IS-DPC	0.93(0.023/0.19/10)			

**Table 6:** Clustering performance on controlled synthetic clustering datasets.

Algorithm	Aggregation			R15		
	ARI	NMI	ACC	ARI	NMI	ACC
FDPAN	0.86(5/4)	0.90(5/4)	0.92(5/4)	0.97(5/4)	0.98(5/4)	0.98(5/4)
RMDPC	0.72	0.88	0.78	0.99	0.99	0.99
DBSCAN-DPC	0.79(10/0.028/5)	0.89(10/0.028/5)	0.72(10/0.028/5)	0.99(10/0.028/5)	0.99(10/0.028/5)	0.99(10/0.028/5)
DPCSA	0.73	0.82	0.90	0.98	0.98	0.99
SMMP	0.99	0.99	0.99	0.99	0.99	0.99
IS-DPC	0.91(0.025/0.18/7)	0.95(0.025/0.18/7)	0.95(0.025/0.18/7)	0.99(0.02/0.13/15)	0.99(0.02/0.13/15)	0.99(0.02/0.13/15)
**Algorithm**	**D31**			**S1**		
	**ARI**	**NMI**	**ACC**	**ARI**	**NMI**	**ACC**
FDPAN	0.35(5/4)	0.75(5/4)	0.39(5/4)	0.71(5/4)	0.87(5/4)	0.73(5/4)
RMDPC	0.93	0.96	0.96	0.53	0.81	0.48
DBSCAN-DPC	0.82(10/0.028/5)	0.93(10/0.028/5)	0.87(10/0.028/5)	0.98(10/0.028/5)	0.98(10/0.028/5)	0.99(10/0.028/5)
DPCSA	0.57	0.69	0.80	0.99	0.99	0.99
SMMP	0.94	0.95	0.96	0.98	0.98	0.99
IS-DPC	0.94(0.02/0.1/31)	0.96(0.02/0.1/31)	0.98(0.02/0.1/31)	0.99(0.02/0.1/15)	0.99(0.02/0.1/15)	0.99(0.02/0.1/15)
**Algorithm**	**S4**			**A3**		
	**ARI**	**NMI**	**ACC**	**ARI**	**NMI**	**ACC**
FDPAN	0.63(6/2)	0.73(6/2)	0.80(6/2)	0.93(10/4)	0.98(10/4)	0.94(10/4)
RMDPC	0.61	0.72	0.79	0.98	0.99	0.99
DBSCAN-DPC	0.63(9/0.028/3)	0.71(9/0.028/3)	0.78(9/0.028/3)	0.98(10/0.028/4)	0.98(10/0.028/4)	0.98(10/0.028/4)
DPCSA	0.61	0.70	0.72	0.95	0.98	0.99
SMMP	0.62	0.72	0.79	0.98	0.98	0.99
IS-DPC	0.63(0.01/0.14/15)	0.73(0.01/0.14/15)	0.80(0.01/0.14/15)	0.98(0.01/0.18/50)	0.99(0.01/0.18/50)	0.99(0.01/0.18/50)
**Algorithm**	**Unbalance**					
	**ARI**	**NMI**	**ACC**			
FDPAN	0.99(5/4)	0.96(5/4)	0.95(5/4)			
RMDPC	1	1	1			
DBSCAN-DPC	0.99(9/0.028/3)	0.99(9/0.028/3)	0.99(9/0.028/3)			
DPCSA	1	1	1			
SMMP	1	1	1			
IS-DPC	0.88(0.01/0.01/8)	0.92(0.01/0.01/8)	0.84(0.01/0.01/8)			

**Table 7:** Clustering performance comparison between original IS-DPC, MOS-ISDPC, and MOS-DPC on synthetic clustering datasets.

Algorithm	Aggregation			R15		
	ARI	NMI	ACC	ARI	NMI	ACC
IS-DPC	0.91	0.95	0.95	0.99	0.99	0.99
MOS-ISDPC	0.81	0.80	0.90	0.82	0.89	0.91
MOS-DPC	0.81	0.81	0.91	0.85	0.91	0.93
**Algorithm**	**D31**			**S1**		
	**ARI**	**NMI**	**ACC**	**ARI**	**NMI**	**ACC**
IS-DPC	0.94	0.96	0.98	0.99	0.99	0.99
MOS-ISDPC	0.79	0.88	0.89	0.82	0.88	0.90
MOS-DPC	0.78	0.87	0.88	0.82	0.88	0.90
**Algorithm**	**S4**			**A3**		
	**ARI**	**NMI**	**ACC**	**ARI**	**NMI**	**ACC**
IS-DPC	0.63	0.73	0.80	0.98	0.99	0.99
MOS-ISDPC	0.54	0.64	0.74	0.81	0.91	0.90
MOS-DPC	0.54	0.63	0.74	0.81	0.91	0.92
**Algorithm**	**Unbalance**					
	**ARI**	**NMI**	**ACC**			
IS-DPC	0.88	0.92	0.84			
MOS-ISDPC	0.54	0.64	0.74			
MOS-DPC	0.92	0.86	0.91			

**Table 8:** Comparison of clustering metrics on the Olivetti Face dataset.

Algorithm	ARI	NMI
FDPAN	0.4029(2/3)	0.7142(2/3)
RMDPC	0.3660(/)	0.7006(/)
DPCSA	0.3352(/)	0.6523(/)
DPC-DBSCAN	0.2809(10/0.02/5)	0.6198(10/0.02/5)
SMMP	0.2268(/)	0.5388(/)
IS-DPC (Proposed)	0.5267(0.99/8/10)	0.7509(0.99/8/10)

## Data Availability

Data are available at https://github.com/DENGZILONG0712/IHS-DPC (accessed on 19 August 2026).

## Abbreviations

The following abbreviations are used in this manuscript:

DPC	Density Peak Clustering
IS-DPC	Iterative Screening Density Peak Clustering algorithm
